# “Pseudo coffee bean sign” without sigmoid volvulus: A case report and literature review

**DOI:** 10.1097/MD.0000000000048513

**Published:** 2026-04-24

**Authors:** Ping-Bin Lin, Min Kong, Jun Chen, Hao Sun

**Affiliations:** a Department of General Surgery, The Central Hospital of Shangrao, Shangrao, Jiangxi Province, China; b Department of Clinical Medicine, Jiangxi Medical College, Shangrao, Jiangxi Province, China.

**Keywords:** coffee bean sign, plain abdominal radiographs, sigmoid volvulus

## Abstract

**Rationale:**

Sigmoid volvulus is a common acute abdominal condition in clinical practice. Delayed treatment can lead to serious consequences. Abdominal plain radiography is an important diagnostic modality for this condition, with the “coffee bean sign” representing its classic radiological finding. However, a “pseudo coffee bean sign” without evidence of intestinal obstruction may also be encountered clinically, necessitating further differential diagnosis.

**Patient concerns:**

We report a 72-year-old male admitted with “left lumbar pain lasting 8 hours.” Comprehensive computed tomography (CT) revealed left ureteral calculi with associated left-sided hydronephrosis. Urinalysis showed 67 white blood cells per microliter (reference range: 0–15/μL).

**Diagnoses:**

Left ureteral calculi with hydronephrosis and urinary tract infection.

**Interventions:**

The patient underwent left ureteroscopic lithotripsy (URS). On postoperative day 1, a follow-up kidney–ureter–bladder (KUB) radiograph revealed the sigmoid colon dilation, consistent with the “coffee bean sign.” The radiology department reported this as a critical finding.

**Outcomes:**

Further comprehensive abdominal CT revealed sigmoid colon redundancy and gaseous distension, with no evidence of sigmoid volvulus or necrosis. At the 1-month follow-up after discharge, the patient remained asymptomatic.

**Lessons:**

The “coffee bean sign” is a classic radiological finding in sigmoid volvulus; however, it may also be present in a small number of patients with sigmoid colon redundancy. When plain abdominal films are inconclusive, CT or barium enema should be promptly performed to rule out intestinal obstruction.

## 1. Introduction

Sigmoid volvulus (SV) is a rare form of intestinal obstruction in clinical practice. Acute sigmoid volvulus is characterized by acute onset, rapid progression, and multiple complications that necessitate prompt intervention. Delayed management can lead to intestinal vascular occlusion, intestinal ischemia and necrosis, acute peritonitis, and in severe cases, intestinal perforation, which can be life-threatening. Therefore, a timely diagnosis is of particular importance. The “coffee bean sign” is a typical finding on abdominal plain films in sigmoid volvulus, although it may also be observed in a small number of otherwise healthy individuals under special circumstances, such as during fasting. We report the case of a 72‑year‑old male with a redundant sigmoid colon who exhibited a “pseudo coffee bean sign” on postoperative abdominal radiography, characterized by gas‑filled bowel loops without evidence of mesenteric torsion, whirl sign, or transition point. The details are as follows.

## 2. Case introduction

**Figure 1.**
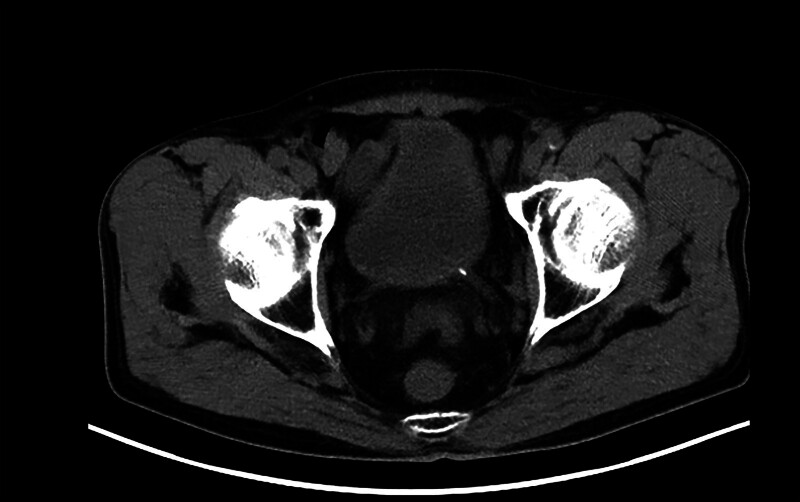
A CT scan of the urinary tract revealed a left ureteral stone measuring approximately 6 mm × 4 mm. CT = computed tomography.

**Figure 2.**
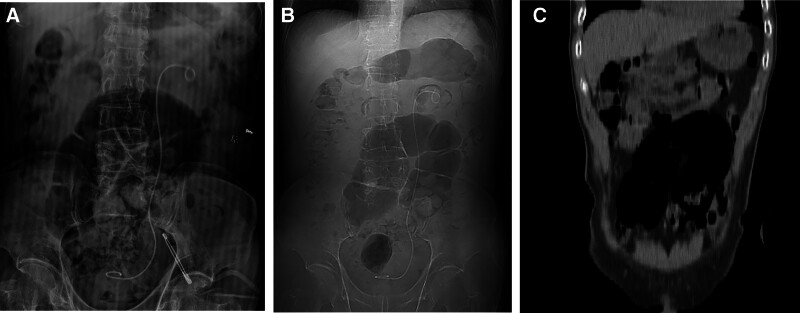
(A) An abdominal plain radiograph demonstrated an inverted U-shaped distended sigmoid colon with a maximum diameter of approximately 7 cm, exhibiting the classic “coffee bean sign.” (B) Scout view from a CT scan. (C) Coronal plane reconstruction and 3D reconstruction, revealing “coffee bean sign.” CT = computed tomography.

**Figure 3.**
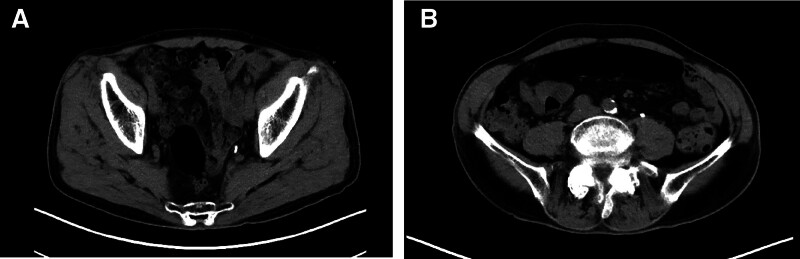
(A, B) CT scan revealed that a redundant sigmoid colon was distended. CT = computed tomography.

A 72-year-old male presented to our hospital with left lumbar pain for 8 hours. Eight hours before admission, the patient developed acute left lumbar pain without obvious precipitating factors, accompanied by gross hematuria. He denied having fever, abdominal distension, nausea, or vomiting. His medical history was notable for constipation, with no prior abdominal surgery. On admission, physical examination revealed no abdominal guarding, tenderness, rebound tenderness, or left costovertebral angle percussion tenderness. The patient was admitted to our hospital for emergency management. Urinalysis revealed 67 white blood cells/μL (reference range: 0–15/μL) and 2979 red blood cells/μL (reference range: 0–15/μL). Antibiotic therapy with ceftazidime was then initiated. Abdominal computed tomography (CT) revealed a lower ureteral calculus, measuring approximately 6 mm × 4 mm (Fig. 1). Cardiopulmonary function was unremarkable. The patient was counseled about his condition and treatment options, including conservative management, extracorporeal shock wave lithotripsy, and surgical intervention, and opted for surgical treatment. Transurethral left ureteroscopic lithotripsy was performed under spinal anesthesia. On postoperative day 1, a kidney–ureter–bladder (KUB) was performed to evaluate stone clearance and double-J stent position. The findings included a left-sided double-J stent in situ, with intestinal gas and dilation in the mid-to-lower abdomen (maximum diameter approximately 7 cm). A classic inverted U-shaped configuration of the sigmoid colon was noted, exhibiting a “coffee bean”-like appearance (Fig. 2). This was initially interpreted as consistent with sigmoid volvulus. The radiology department reported this as a critical value and notified the attending physician of it. Upon evaluation, the patient denied abdominal pain, distension, nausea, or vomiting. Repeat physical examination revealed no abdominal peristalsis, guarding, tenderness, or rebound tenderness, with normal bowel sounds. To clarify the diagnosis, a comprehensive abdominal CT was performed, which revealed elongation and dilation of the sigmoid colon without obstructive features (maximum sigmoid diameter approximately 7 cm, absence of whirl sign, transition point, and mesenteric vessel crowding). The sigmoid volvulus was excluded (Fig. 3). The patient remained asymptomatic postoperatively and was discharged on postoperative day 3. At the 1-month follow-up after discharge, the patient remained asymptomatic.

## 3. Discussion

Sigmoid volvulus is a distinct form of closed-loop intestinal obstruction. Based on the speed of onset, it is classified into subacute and acute fulminant types. The acute fulminant subtype rapidly progresses and requires emergency treatment. The global incidence of sigmoid volvulus varies owing to differences in dietary habits, geographical altitude, and ethnicity. High-incidence regions include Africa, the Middle East, and South Asia, whereas North America, Western Europe, and Australia have lower rates.^[1]^ It ranks as the third leading cause of colonic obstruction, accounting for approximately 25% to 50% of all cases, with an annual incidence of 1 to 2 per 1,00,000 population.^[2]^ Sigmoid volvulus has a multifactorial etiology and predominantly affects middle-aged and elderly men aged 40 to 80 years.^[3]^ Clinical manifestations are often influenced by factors such as advanced age, chronic constipation, diabetes, neurological disorders, prior abdominal surgery, and megacolon.^[4]^ Pathologically, the sigmoid loop rotates around the mesentery and causes intestinal obstruction. This leads to occlusion of the venous and arterial supply to the affected segment, followed by rapid distension of the closed loop, resulting in intestinal wall ischemia and necrosis and, in severe cases, intestinal perforation.

For acute sigmoid volvulus, timely diagnosis and early intervention are critical for reducing associated complications. A comprehensive assessment should integrate the patient’s clinical manifestations, physical examination findings, laboratory results, and imaging or endoscopic studies, including abdominal radiography, barium enema, CT, and endoscopy.

Clinical presentation typically includes the following features:

*Abdominal pain*: One of the most characteristic symptoms is predominantly localized to the left lower quadrant or is diffuse, persistent, and often exacerbated by paroxysms.
*Abdominal distension*: Asymmetric, with prominence in the left lower quadrant.
*Complete obstruction*: May manifest as absolute constipation (cessation of bowel movements and flatus). Some patients may also present with small amounts of bloody mucoid stool, nausea, or vomiting.

Physical examination findings include:

*Inspection*: Abdominal distension, with visible “intestinal loops” (or “peristaltic waves”) in the left lower quadrant.
*Palpation*: Classic signs of acute peritonitis may be present, including abdominal guarding, tenderness, and rebound tenderness. Occasionally, distended bowel loops may be palpable.
*Auscultation*: Bowel sounds may initially be hyperactive and accompanied by borborygmi (rushes) or high-pitched sounds. In later stages, paralytic ileus may develop because of toxin absorption, resulting in diminished bowel sounds.

Abdominal radiography is the first-line imaging modality for diagnosing sigmoid volvulus, with a diagnostic yield of 57% to 90%.^[5]^ It demonstrates the sigmoid colon dilation and distension, with characteristic findings including the “coffee bean sign,” “rectal gaslessness sign,” “double-loop sign,” “the liver overlap sign,^[6]^” and “the northern exposure sign.^[7,8]^”

The “coffee bean sign” is a classic x-ray finding for acute sigmoid volvulus. It arises from sigmoid twisting, resulting in closed-loop obstruction with marked distension of the involved loop, resembling a coffee bean, where the central indentation corresponds to the apposed walls of the distended loop and the outer contour reflects the outer intestinal wall. This sign is observable in approximately 60% of cases and is one of the most sensitive findings on abdominal radiography, with a reported sensitivity of 60% to 80%.^[9]^ However, the “coffee bean sign” is not pathognomonic for sigmoid volvulus; it may also occur in other conditions such as simple distal colonic obstruction,^[10]^ transverse colon or small bowel distension compressing the sigmoid loop, or in patients with an elongated sigmoid colon.^[9]^ The sigmoid colon, a segment of the large intestine with significant anatomical variability,^[11]^ has an average length of approximately 40 cm. An elongated sigmoid colon – exceeding this length – is a congenital malformation resulting from abnormal sigmoid development, representing a rare chronic condition. In certain circumstances, such patients may also exhibit the “coffee bean sign” on abdominal radiography. Additional imaging modalities can aid confirmation when abdominal radiography yields inconclusive results. Barium enema reveals the characteristic “bird beak sign” at the obstruction site,^[12]^ where barium tapers gradually terminate at the twisted segment, resembling a bird’s beak, improving diagnostic yield by 20% to 30%.^[13]^ It also helps exclude primary intestinal tumors and clarify the cause of obstruction. CT is considered the gold standard and key diagnostic method for sigmoid volvulus, with 100% sensitivity and >90% specificity.^[14]^ Beyond confirming the diagnosis, CT precisely and promptly characterizes the obstruction site to guide clinical management. It clearly demonstrates volvulus-specific findings, including sigmoid twisting around the mesentery and vasculature, narrowing of the afferent and efferent loops, and signs of strangulation. Critical features for identifying obstruction presence and level include the “whirl sign,” “‘X’ marks the spot sign,^[13]^” “split wall sign,” and “ steel pan sign.” Colonoscopy serves both diagnostic and therapeutic purposes, and in appropriate cases, detorsion can be attempted. It offers advantages such as short procedure time and rapid resolution. In summary, for patients, once clinical manifestations or signs of peritoneal irritation are observed, or if abdominal x-ray films show atypical findings, a comprehensive abdominal CT scan should be performed immediately to assess intestinal viability. If the CT scan reveals no evidence of intestinal necrosis and the patient’s vital signs are stable, endoscopic repositioning is the preferred nonsurgical treatment option.^[15]^ However, emergency surgery is indicated if endoscopic reduction fails, imaging shows intestinal necrosis or perforation, or the patient presents with acute peritonitis and unstable vital signs.^[16]^

In this case, sigmoid colon distension was caused by the elongation of the sigmoid colon. Clinically, it is necessary to rule out other potential causes of postoperative sigmoid colon distension and dilation, as follows:

1. Postoperative intestinal obstruction: One of the common complications of abdominal surgery, the activation of intestinal μ-receptors by opioid drugs, is a key inducement.^[17]^ It typically occurs in the early postoperative period (1–3 days) and involves the entire intestine, predominantly the small intestine, although the colon may also have gas retention. Abdominal plain films show multiple air-fluid levels, and gas shadows are also present in the colon.
2. Ogilvie’s syndrome (acute colonic pseudo-obstruction): A secondary colonic dilation disorder without mechanical obstruction factors,^[4]^ and a common induction following major abdominal surgery.^[18]^ Patients often present with marked abdominal distension but relatively mild abdominal pain. Radiographic examination revealed colonic dilation, mainly with gas accumulation, and CT examination was helpful in excluding organic lesions.
3. Gas dilation caused by fasting: A benign condition. Patients were in a fasting state, with abdominal distension but no nausea or vomiting, and normal bowel sounds. Abdominal plain films show diffuse increased gas in the gastrointestinal tract, without isolated, progressive dilation of specific intestinal segments or air-fluid levels.

This elderly male patient required surgery for a left ureteral stone. Preoperative and postoperative fasting (including nil by mouth) was performed. On postoperative day 1, abdominal plain radiography revealed the “coffee bean” sign. Further CT examination confirmed sigmoid colon redundancy and gaseous distension, with no evidence of sigmoid volvulus. The patient was discharged 2 days later without abdominal discomfort. However, this case has a limitation in that no follow-up abdominal CT was performed prior to discharge, as early-stage volvulus cannot be completely ruled out.

A review of the relevant literature and clinical experience suggests that the patient’s radiograph differs from the classic x-ray findings of sigmoid volvulus in the following aspects:

1. In classic cases, extreme gaseous distension in the sigmoid volvulus typically results in a smooth intestinal wall with loss of normal mucosal architecture on the “coffee bean sign.^[19]^” In contrast, the bowel loops on this patient’s abdominal plain film showed segmental intestinal tubes.
2. Sigmoid volvulus may present with air-fluid levels in the bowel lumen (manifesting as a “double-loop sign”), with the sigmoid colon overlapping the liver and extending cephalad toward the transverse colon. However, this patient lacked the “ double-loop sign,” “ the liver overlap sign,” or “the northern exposure sign.”
3. The current radiograph only demonstrated characteristic gaseous distension of the sigmoid colon, with no notable gas visualized in other intestinal segments.

## 4. Conclusion

In conclusion, sigmoid volvulus constitutes an acute abdominal emergency that requires urgent intervention. While the “coffee bean sign” is a characteristic finding of sigmoid volvulus on abdominal plain radiography, it may also be observed in fasting patients or in those with a normally elongated sigmoid colon. When abdominal plain films are diagnostically inconclusive, further evaluations, such as barium enema and CT, should be promptly performed to exclude intestinal obstruction.

## Author contributions

**Funding acquisition:** Hao Sun.

**Investigation:** Min Kong, Jun Chen.

**Resources:** Min Kong, Jun Chen.

**Writing – original draft:** Ping-Bin Lin, Hao Sun.

**Writing – review & editing:** Ping-Bin Lin, Hao Sun.
